# Associations between Environmental Tobacco Smoke Exposure in Early Life and Astigmatism among Chinese Preschool Children

**DOI:** 10.3390/ijerph16193725

**Published:** 2019-10-03

**Authors:** Chen-Guang Li, Gui-You Yang, Katrina L. Schmid, Li-Hua Huang, Guan-Hao He, Li Liu, Zeng-Liang Ruan, Wei-Qing Chen

**Affiliations:** 1 Department of Biostatistics and Epidemiology, School of Public Health, Sun Yat-sen University, Guangzhou 510080, China; lichg3@mail2.sysu.edu.cn (C.-G.L.); yanggy7@mail2.sysu.edu.cn (G.-Y.Y.); hlihua2@mail2.sysu.edu.cn (L.-H.H.); heguanh@mail2.sysu.edu.cn (G.-H.H.); liuli9931@163.com (L.L.); ruanzliang@mail2.sysu.edu.cn (Z.-L.R.); 2 School of Optometry and Vision Science, Faculty of Health, Queensland University of Technology, 60 Musk Ave, Kelvin Grove, Brisbane, QLD 4059, Australia; k.schmid@qut.edu.au; 3 Department of Information Management, Xinhua College of Sun Yat-sen University, Guangzhou 510080, China

**Keywords:** environmental tobacco smoke, astigmatism, preschool children, early life

## Abstract

This study aimed to investigate the association between environmental exposure to tobacco smoke (ETS) during early life and astigmatism in Chinese preschool children. In this cross-sectional study, information concerning prenatal and postnatal ETS exposure at three stages of early life (during pregnancy, from birth to one year and from one to three years), visual problems of children and parents (including a confirmed diagnosis of astigmatism), socio-demographics and perinatal characteristics were obtained from 27,890 parent-reported questionnaires. Logistic regression analyses were undertaken to yield adjusted odds ratios (OR) for assessing their associations. After adjusting for the potential confounders, children were more likely to exhibit astigmatism when they were exposed to ETS during pregnancy + from one to three years [OR (95% CI) = 1.37 (1.02, 1.84)], or from birth to one year + from one to three years [OR (95% CI) = 1.36 (1.11, 1.66)], or during pregnancy + from birth to one year + from one to three years old [OR (95% CI) = 1.29 (1.16, 1.45)], compared to children without ETS exposure at any stage of early life. In Chinese preschool children, prenatal and postnatal astigmatism was associated with ETS exposure; the greater the ETS dose, the greater the astigmatism risk.

## 1. Introduction

Astigmatism is a common visual problem resulting from the unequal meridional curvatures of one or more refractive surfaces of the eye (cornea and/or lens), leading to blurred vision at all distances if not corrected [1,2]. The prevalence of astigmatism in Chinese preschool children from Shanghai has been noted to be generally higher (ranging from 5.8% to 19.8% in children aged three to six years) [3], than that reported in Western countries (ranging from 7.6% to 8.1% in the Multiethnic Pediatric Eye Disease Study (MEPEDS) and the Baltimore Pediatric Eye Disease Study (BPEDS) of children aged two to six years) [4]. High degrees of astigmatism in early life can induce meridional amblyopia [5,6], it is associated with the development of myopia [5,7,8] and thus impacts normal visual development. This warrants the identification of modifiable factors that increase the risk of astigmatism and the development of appropriate public health messages.

In addition to hereditary factors [9], numerous lines of evidence demonstrate that environmental factors play crucial roles in ocular growth and refractive development, particularly exposure to risk factors in early life, such as feeding pattern, birth weight, exposure to toxic substances including nicotine [10,11,12]. Based on the premise that nicotine from tobacco smoke influences refractive development via activating nicotinic acetylcholine receptors in the eye [4,9,13,14,15,16], the impact of maternal active smoking is well-established. Previous work has emphasized myopia, hypermetropia, strabismus and amblyopia, rather than astigmatism [17]. Most studies have been conducted in Western developed countries and Singapore, where the maternal active smoking rate was usually high and socio-demographics differed substantially from those in developing countries including China. The sample size has been usually less than 8000 children [10]. None of the studies have reported the relative sensitivity of different developmental stages within the most critical period for refractive development, which is usually considered to be up to three-years of age [18,19,20].

It has been proposed that exposure to tobacco smoke (ETS) exposure, i.e., passive smoking, which is formed by a mixture of sidestream smoke (around 80%) and mainstream smoke has similar effects to maternal active smoking [21,22], and may be even more toxic [23,24]. The potential for ETS exposure to increase the risk of early-onset refractive error is not well established. In China, less than 2% of women smoke [25], but approximately 44% are exposed to ETS for at least 15 min per day on at least one day per week [26]. Thus, the aim was to investigate the association between exposure to ETS and early-onset astigmatism in Chinese preschool children, and determine the most critical period for exposure related risk.

## 2. Materials and Methods

### 2.1. Study Population

A total of 29,595 children aged 2 to 7 years from 171 kindergartens in Longhua District of Shenzhen of China were enrolled. In October, 2017 their primary caregivers were asked to complete online a self-administered structured questionnaire guided by trained kindergarten teachers. Questionnaires were excluded if: (1) The mother reported being an active smoker before or during pregnancy; (2) it was reported that the child had a visual problem other than astigmatism; and (3) there was missing information regarding ETS exposure or eye conditions. Thus 27,890 (94.2%) questionnaires were included for analysis.

The study was conducted in accordance with the Declaration of Helsinki, and the protocol was approved by the Ethics Committee of the School of Public Health at Sun Yat-sen University (ethics clearance No.: 2015-016), the local Women’s and Children’s Hospital and the Administration of Education. Informed consent was obtained from the primary caregivers.

### 2.2. Data Collection

Data were collected using a self-administered structured questionnaire including information about parental socio-demographic characteristics (i.e., age at childbirth, education, family monthly income, and history of common visual problems including astigmatism) and children’s general information (i.e., date of birth, gender, birth weight, feeding pattern, premature birth, electronic screens exposure, prenatal and postnatal ETS exposure, and the child’s history of common visual problems including astigmatism).

### 2.3. Assessment of ETS Exposure

Children’s prenatal and postnatal ETS exposure in the three stages of early life [pregnancy (S1), from birth to 1 year (S2) and from 1 to 3 years (S3)] was determined by the following questions: (1) Did any household members ever smoke at home? (0 score = ‘no’, 1 = ‘yes’); and (2) If yes: How many cigarettes (of the conventional type) did they smoke per day at home? (1 score = ‘1–5 cigarettes’, 2 = ‘6–10 cigarettes’, 3 = ‘11–15 cigarettes’, 4 = ‘16–20 cigarettes’, 5 = ‘>20 cigarettes’); and (3) how long was the pregnant mother (S1) or the child (S2 and S3) exposed to ETS per day? (1 score = ‘1–15 min’, 2 = ‘16–30 min’, 3 = ‘31–45 min’, 4 = ‘46–60 min’, 5 = ‘61–90 min’, 6 = ‘91–120 min’, 7 = ‘>120 min’). Eight categories were used to classify the ETS exposure conditions during each of the three stages (‘yes’ or ‘no’).

To assess the dose-response relationship between ETS exposure and astigmatism, the total score of ETS exposure based on the number or the time of exposing cigarettes smoked per day was respectively calculated. The total score of ETS exposure based on the number of exposing cigarettes smoked per day (TSETS-NECSPD) was the sum of each stage’s cigarette score which was calculated as in question (2) above. For example, if a child’s whole ETS exposure was 6–10 cigarettes/day during pregnancy (S1), 11–15 cigarettes/day from birth to 1 year (S2) and 1–5 cigarettes/day from 1 to 3 years (S3) respectively, the child’s TSETS-NECSPD was “2 + 3 + 1 = 6”. While the calculation of the total score of ETS exposure based on the time of exposing cigarettes smoked per day (TSETS-TECSPD) was the same as TSETS-NECSPD according to question (3) above. The subsequent classification of TSETS-NECSPD (shown in Table 2) and TSETS-TECSPD (shown in Table 3) was mainly based on a balanced sample size.

### 2.4. Presence of Astigmatism

The following questions were asked about the child’s eye conditions: (1) Has your child ever been diagnosed as having poor sight by the oculist? (0 = ‘no’, 1 = ‘yes’, 2 = ‘uncertain’); and if yes, the subsequent questions were asked separately, i.e., questions (2)–(7): Has your child ever been diagnosed as having astigmatism/myopia/hyperopia/strabismus/amblyopia/other common visual problems? (0 = ‘no’, 1 = ‘yes’, 2 = ‘uncertain’). In the current study, only questionnaires where it was reported that children had either no visual problems or the only reported visual problem was astigmatism were included in the analysis.

### 2.5. Confounding Variables

The following confounding covariates were first chosen based on the published literature [10,11,12,27], including children’s gender and age, parental education level, family monthly income, parental age at childbirth, feeding pattern, electronic screen exposure and parental history of astigmatism. A directed acyclic graph (DAG, Figure S1) was then constructed based on the existing literature to select a minimally sufficient set of covariates to adjust for confounding variables [28] (DAGitty v2.3 software, www.dagitty.net). Based on the DAG, the following variables were retained as confounders in the statistical models: Age, parental education level, family monthly income, parental age at childbirth and electronic screen exposure (Figure S2).

### 2.6. Data Analysis

The baseline characteristics of children with and without astigmatism were compared using chi-squared tests for categorical variables. Logistic regression was used to calculate odds ratios (ORs) and 95% confidence intervals (95% CIs) to evaluate the association between the combinations of prenatal and postnatal ETS exposure and astigmatism, as well as the dose-response relationship, with adjustment for confounders. Tests for linear trends involved entering the median value of each category of cigarette score and time score as a continuous variable in the models.

With reference to the literature [9], sensitivity analyses were conducted among children whose parents were reported to have normal vision (*n* = 15,848) and other visual problems except for astigmatism (*n* = 6896), respectively. The analyses were applied to assess the associations between ETS exposure and astigmatism among parents without any visual problems and with other visual problems, while including the adjustment for confounders. The effect of birth weight and history of premature birth were also evaluated, as was the interaction between hereditary factors and ETS exposure.

The statistical analysis was carried out using a commercially available software package (SPSS for Windows, version 23.0; IBM-SPSS, Chicago, IL, USA). Statistical analyses were two-sided; a *p*-value of < 0.05 was considered statistically significant.

## 3. Results

### 3.1. Comparison of Children’s Baseline Characteristics

Of the 27,890 children whose questionnaires were included in this study, 2022 (7.2%) included a diagnosis of astigmatism. The children were aged between 2.0 and 7.7 years (mean age = 4.6 ± 0.9 years), and approximately half were boys (male: 54.2%). Table 1 presents the baseline characteristics of the children. Children with and without astigmatism, differed in age and electronic screen exposure; however, other characteristics were similar, including gender distribution, parental education level, family monthly income and parental age at childbirth.

### 3.2. Association of Astigmatism with Combinations of Prenatal and Postnatal ETS Exposure

**Figure 1:**
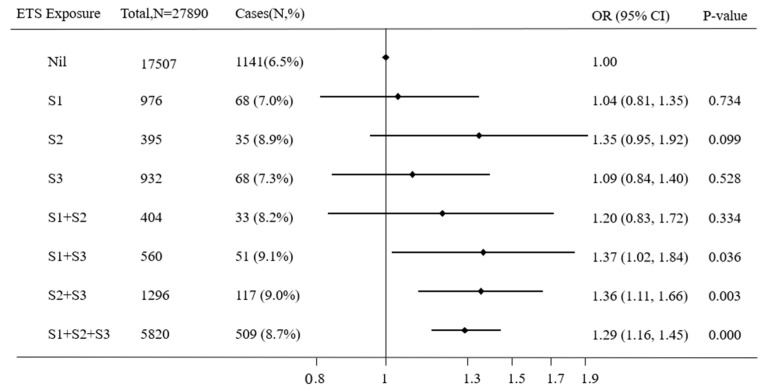
The associations of astigmatism with combinations of pre- and post-natal ETS exposure. ETS: Environmental tobacco smoke, OR: Odds ratios, S1: During pregnancy, S2: From birth to one year, S3: From one to three years. A binary logistic regression model was used, while adjusting for children’s gender and age, parental education level, family monthly income, parental age at childbirth, and electronic screen exposure.

To determine the critical period of ETS exposure increasing the risk of astigmatism, the link between astigmatism and ETS for different developmental stages was analyzed (Figure 1). Among all of the eight subgroups, compared with non-exposed children during all stages of early life, only the children who were exposed to ETS from one to three years (S3), together with ETS exposure during pregnancy (S1) (adjusted OR = 1.37, 95% CI = 1.02–1.84, *p* = 0.036) or from birth to one year (S2) (adjusted OR = 1.36, 95% CI = 1.11–1.66, *p* = 0.003) or both during pregnancy (S1) and from birth to one year (S2) (adjusted OR = 1.29, 95% CI = 1.16–1.45, *p* < 0.001), had a significantly higher risk of astigmatism after controlling for all confounding variables. Children who were exposed to ETS in only one of the three stages [i.e., during pregnancy (S1) or from birth to one year (S2) or from one to three years (S3)] or only the previous two stages [i.e., exposure during pregnancy (S1) and from birth to one year (S2)] did not show a significantly increased risk of astigmatism. However, notably, when children were exposed to ETS from birth to one year (S2) only, the OR obviously upgraded to a higher level and reached borderline significance (OR = 1.35, 95% CI = 0.95–1.92, *p* = 0.099).

The risk of astigmatism increased as the exposure score increased according to the number of exposing smoked cigarettes per day, the adjusted ORs were: 1.19 (95% CI = 0.98–1.45) for 1 score, 1.25 (95% CI = 1.10–1.41) for 2–3 scores; 1.30 (95% CI = 1.11–1.52) for 4–7 scores, 1.40 (95% CI = 1.12–1.76) for 8–11 scores and 1.19 (95% CI = 0.86–1.64) for ≥12 scores, respectively; and the trend test was significant (*p* for trend < 0.001) (Table 2).

The dose-response relationship was also observed between the score for the time of exposing ETS per day from gestation to three years, and the adjusted ORs were: 1.15 (95% CI = 0.97–1.38) for 1 score, 1.28 (95% CI = 1.15–1.43) for 2–3 scores, 1.35 (95% CI = 1.06–1.71) for 4–5 scores, 1.40 (95% CI = 1.07–1.82) for 6–8 scores and 1.12 (95% CI = 0.78–1.60) for ≥9 scores, respectively; and the trend test was significant (*p* for trend < 0.001) (Table 3).

### 3.3. Sensitivity Analysis

**Figure 2:**
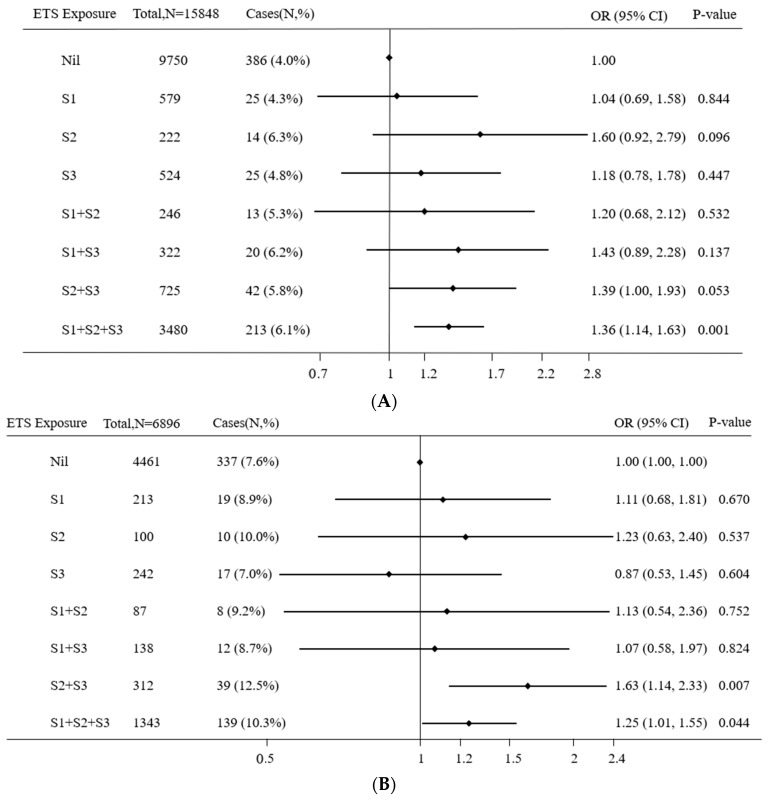
Sensitivity analysis of association between astigmatism and combinations of pre- and post-natal ETS exposure without any parental history of visual problems (**A**) and with parental history of others visual problems except for astigmatism (**B**). ETS: Environmental tobacco smoke, OR: Odds ratios, S1: During pregnancy, S2: From birth to one year, S3: From one to three years. A binary logistic regression model was used, while adjusting for children’s gender and age, parental education level, family monthly income, parental age at childbirth and electronic screen exposure.

Among the children whose parents reported no visual problems, compared with non-exposed children during all the early stage of life, children with sustained ETS exposure from pregnancy to three years (adjusted OR = 1.36, 95% CI = 1.14–1.63, *p* < 0.001) had the higher risk of astigmatism after controlling for all confounding variables (Figure 2A). The results among the children whose parents reported other visual problems except for astigmatism were similar: Children with ETS exposure in the two stages from birth to one year and from one to three years (adjusted OR = 1.63, 95% CI = 1.14–2.33, *p* = 0.007) and sustained ETS exposure from pregnancy to three years (adjusted OR = 1.25, 95% CI = 1.01–1.55, *p* = 0.044) had the higher risk of astigmatism compared with non-exposed children after controlling for confounders (Figure 2B).

Among the children with normal birth weight, compared with non-exposed children during all early stages of life, children with ETS exposure in the two stages from birth to one year and from one to three years (adjusted OR = 1.36, 95% CI = 1.09–1.69, *p* = 0.006) and sustained ETS exposure from pregnancy to three years (adjusted OR = 1.29, 95% CI = 1.14–1.46, *p* < 0.001) had the higher risk of astigmatism after controlling for confounders. However, only the children who were exposed to ETS during pregnancy together with from one to three years (adjusted OR = 3.98, 95% CI = 1.16–13.6, *p* = 0.028) had a significantly higher risk of astigmatism after controlling for confounders compared with non-exposed children during all the early stage of life (Table 4).

Among the children reported as full-term children with ETS exposure in the two stages from birth to one year and from one to three years (adjusted OR = 1.35, 95% CI = 1.09–1.67, *p* = 0.006) and sustained ETS exposure from pregnancy to 3 years (adjusted OR = 1.30, 95% CI = 1.15–1.46, *p* < 0.001) had the higher risk of astigmatism compared with non-exposed children during all the early stage of life after controlling for confounders. However, no significant difference in risk was found among premature infant (Table 5).

### 3.4. Interaction Analysis

The results showed that there was no interaction between hereditary factors and pre- and post-natal ETS exposure on astigmatism (adjusted OR = 0.88, 95% CI = 0.72–1.06) (Table 6).

## 4. Discussion

This large-scale study undertaken in Chinese preschool-aged children showed that both prenatal and postnatal ETS exposure increased the risk of early-onset astigmatism. ETS exposure during one to three years (S3) together with at least one additional stage [pregnancy (S1) or from birth to one year (S2)] or exposure during all three stages significantly increased the risk of astigmatism, which suggests that the detrimental effect of prenatal and postnatal ETS exposure accumulates. Further analysis of the dose-response relationship showed that the risk of astigmatism significantly increased with increasing daily cigarette consumption by household members and overall early-life ETS exposure score.

Past studies have primarily focused on the effects of maternal active smoking on their children’s refractive errors [17]. A meta-analysis which was stratified by different types of refractive errors demonstrated that children whose mother smoked during pregnancy were 1.47 (95% CI = 1.12–1.93) times and 1.43 (95% CI = 1.23–1.66) times more likely to suffer from amblyopia and hyperopia, respectively, compared with children whose mother did not smoke. No statistical difference was found for myopia (OR = 0.59, 95% CI = 0.25–1.38) [10], which was consistent with the findings for Singaporean Chinese children (OR = 0.39, 95% CI = 0.12–1.34, *p* = 0.14) [16]. It was noteworthy to mention that a large study (namely the Multi-Ethnic Pediatric Eye Disease Study (MEPEDs) and the Baltimore Pediatric Eye Disease Study (BPEDS) reported that the occurrence of astigmatism was significantly different between children whose mothers smoked during pregnancy and those whose mothers did not smoke (OR = 1.46, 95% CI = 1.14–1.87) [4].

The finding that ETS can increase the risk of astigmatism is consistent with reports of the impacts of passive smoking on the occurrence of some other refractive errors, although outcomes of these studies are mixed. For example, an American multi-ethnic study found no statistically significant association between passive smoking and any type of anisometropia [29], and a study by Stone et al. [13] indicated a minor protective effect on myopia. In contrast, a recent study based on three-year-old children in Singapore [30] demonstrated that childhood exposure to passive smoke from birth to before six months slightly increased the risk of early-onset myopia, and an Egyptian study involving 300 children between the age of five and 12 years [31] suggested that passive smoking might be associated with a refractive error shift towards hypermetropia.

The finding that the detrimental effect of prenatal and postnatal ETS exposure on astigmatism accumulates suggests the existence of a dose-response relationship. The possibility of a dose-response relationship has rarely been mentioned in the literature. A prospective study in Denmark supported a significant dose-response relationship between maternal smoking and strabismus in children aged six to 13 years (<five cigarettes/day: relative risk (RR) = 0.95, 95% CI = 0.80–1.14; 5–9 cigarettes/day: RR = 1.38, 95% CI = 1.12–1.70; ≥10 cigarettes/day: RR = 1.90, 95% CI = 1.57–2.30) [32]. However, a dose-response relationship was not found between maternal smoking and amblyopia in Singapore Chinese preschool children [16]. The potential biological mechanism for the observed refractive error associations reported here and in previous studies [13,16], may be related to the presence of nicotinic acetylcholine receptors (nAChRs) in the retina [31]. Neuronal nAChRs play an important role in neuronal migration and growth cone direction. Exposure to nicotine in cigarette smoke may activate nAChRs in the occipital cortex, which may induce shifts in refraction, including the development of astigmatism [4,15,33]. Findings from animal models also support the involvement of nAChRs [14,34]. Moreover, some studies identified that a toxic prenatal environment may lead to disturbances in the neural growth of the cones and in optic disc hypoplasia due to the reduction of placental flood flow [35]. Furthermore, a recent study showed that cigarette smoking is associated with decreased choroidal vascularity and this association appears to be dose dependent [36]. As ETS decreases blood flow to the eye this might slow early ocular development and thus prevent or slow the reduction of congenital astigmatism.

Several limitations should be acknowledged. First, the reliability and quantitative accuracy of self-reported questionnaire-derived data concerning the amount and duration of early-life ETS exposure was uncertain. Future research that adopts objective methods to assess passive smoke exposure, such as measuring cotinine levels (the predominant metabolite of nicotine) in human biological specimens [23], would eliminate potential self-report bias. However, cotinine concentration can be affected by polymorphisms of the CYP2A6*4 gene (the key phase I enzyme for nicotine metabolism) [37], and thus whether it accurately reflects an infant’s ETS exposure level is unknown. Although the optimal approach of ETS measurement might be repeated by way of biochemical cotinine assessments throughout early life, this is impractical. The primary caregivers of children are likely to pay attention to the smoking environment and are thus are likely to accurately recall the ETS exposure. Second, there is no uniform definition for ETS. Here, ETS was defined as having at least one person who smoked at home, and information about passive smoking in other places was not sought, therefore, this might result in an underestimation of ETS exposure. Third, this study was cross-sectional and the study design did not permit a reliable inference of causality, even though the observed positive association between ETS exposure and early-onset astigmatism could be causal according to the aforementioned biological mechanism. Fourth, it was a pity that information regarding the child’s ETS exposure from three to six years was absent, this data could have provided further evidence for the possible accumulative effects of early-life ETS exposure. Fifth, due to the large sample size in the present study, most of p values were less than 0.005 or 0.001, although ORs were about two indicating the weak association between prenatal and postnatal ETS exposure and astigmatism, so we should cautiously determine the causal relationship between them, and longitudinal studies or animal trials are needed to confirm a causal relationship in the future. Finally, measures of astigmatism were obtained through the retrospective recall of a hospital-confirmed astigmatism diagnosis, and thus, the overall astigmatism prevalence may have been underestimated. This may be a potential source of misclassification bias. The degree of astigmatism was not quantified and thus it may be that with an increased ETS, both the likelihood of astigmatism and its severity increased but this was not assessed here.

## 5. Conclusions

Higher amounts of prenatal and postnatal ETS exposure were associated with a higher risk for early-onset astigmatism. It is likely that the impact of ETS exposure is accumulative; this highlights the importance of limiting an infant’s passive exposure as early as possible. Public health messages should include information about the vision impact of ETS with the aim of reducing early-life ETS exposure. Future studies that adopt birth cohort design, include comprehensive eye examinations, use multi-center samples and employ repeated biochemical assessments are recommended.

## Figures and Tables

**Table 1:** Baseline characteristics of the participating children with (*n* = 2022) and without (*n* = 25,868) astigmatism.

Characteristics	Total *n* = 27,890	Astigmatism (*n*, %)	*χ* ^2^	*p*
Gender			1.16	0.28
Male	15,128	1120 (7.4%)		
Female	12,762	902 (7.1%)		
Age			19.87	0.000
<3 years	554	30 (5.4%)		
3 to <4 years	7701	482 (6.3%)		
4 to <5 years	9570	742 (7.8%)		
5 to <6 years	9200	704 (7.7%)		
≥6 years	865	64 (7.4%)		
Maternal education			0.25	0.88
≤High school	11,923	875 (7.3%)		
Junior college education	10,064	724 (7.2%)		
≥Undergraduate college	5903	423 (7.2%)		
Paternal education			2.80	0.25
≤High school	10,830	789 (7.3%)		
Junior college education	8881	670 (7.5%)		
≥Undergraduate college	8179	563 (6.9%)		
Family monthly income ^a^			8.14	0.09
≤5000 yuan	4098	301 (7.3%)		
5001–10,000 yuan	7352	572 (7.8%)		
10,001–20,000 yuan	9206	668 (7.3%)		
20,001–30,000 yuan	3976	276 (6.9%)		
>30,000 yuan	3258	205 (6.3%)		
Maternal age at childbirth			2.50	0.48
≤22 years	3271	242 (7.4%)		
22 to <30 years	19,232	1408 (7.3%)		
30 to <40 years	5236	358 (6.8%)		
≥40 years	151	14 (9.3%)		
Paternal age at childbirth			4.22	0.24
≤22 years	1143	82 (7.2%)		
22 to <30 years	16,451	1221 (7.4%)		
30 to <40 years	9506	653 (6.9%)		
≥40 years	790	66 (8.4%)		
Electronic screen exposure			139.10	0.000
No	6870	278 (4.0%)		
Yes	21,020	1744 (8.3%)		

^a^ At the exchange rate as of September 2019, 1 yuan is approximately equal to 0.15 U.S. dollars.

**Table 2:** The dose-response relationship between total score of ETS exposure based on the number of exposing cigarettes smoked per day (TSETS-NECSPD) and astigmatism.

TSETS-NECSPD	Total (*n* = 27,890)	Cases (*n*, %)	Crude OR (95% CI)	Adjusted OR ^a^ (95% CI)
0	17,507	1141 (6.5%)	1.00	1.00
1	1547	122 (7.9%)	1.23 (1.01–1.49) *	1.19 (0.98–1.45)
2–3	4892	409 (8.4%)	1.31 (1.16–1.47) ***	1.25 (1.10–1.41) ***
4–7	2472	217 (8.8%)	1.38 (1.19–1.61) ***	1.30 (1.11–1.52) ***
8–11	952	90 (9.5%)	1.50 (1.20–1.88) ***	1.40 (1.12–1.76) **
≥12	520	43 (8.3%)	1.29 (0.94–1.78)	1.19 (0.86–1.64)
*p* for trend			<0.001	<0.001

TSETS-NECSPD: The total score of environmental exposure to tobacco smoke based on the number of exposing cigarettes smoked per day. ^a^ Adjusting for children’s gender and age, parental education level, family monthly income, parental age at childbirth and electronic screen exposure.* *p* < 0.05; ** *p* < 0.005; *** *p* < 0.001.

**Table 3:** The dose-response relationship between the total score of environmental exposure to tobacco smoke based on the time of exposing cigarettes smoked per day (TSETS-TECSPD) and astigmatism.

TSETS-TECSPD	Total (*n* = 27,890)	Cases (*n*, %)	Crude OR (95% CI)	Adjusted OR ^a^ (95% CI)
0	17,507	1141 (6.5%)	1.00	1.00
1	2008	154 (7.7%)	1.19 (1.00–1.42) *	1.15 (0.97–1.38)
2–3	6365	548 (8.6%)	1.35 (1.22–1.50) ***	1.28 (1.15–1.43) ***
4–5	889	81 (9.1%)	1.44 (1.14–1.82) **	1.35 (1.06–1.71) **
6–8	678	64 (9.4%)	1.50 (1.15–1.95) **	1.40 (1.07–1.82) **
≥9	443	34 (7.7%)	1.19 (0.84–1.70)	1.12 (0.78–1.60)
*p* for trend			<0.001	<0.001

TSETS-TECSPD: The total score of environmental exposure to tobacco smoke based on the time of exposing cigarettes smoked per day. ^a^ Adjusting for children’s gender and age, parental education level, family monthly income, parental age at childbirth and electronic screens exposure. * *p* < 0.05; ** *p* < 0.005; *** *p* < 0.001.

**Table 4:** Sensitivity analysis of association between astigmatism and combinations of pre- and post-natal ETS exposure among children with normal birth weight and low birth weight.

ETS Exposure			Subgroup 1 AOR ^a^ (95% CI)	Subgroup 2 AOR ^a^ (95% CI)
S1	S2	S3		
No	No	No	1.00	1.00
Yes	No	No	1.01 (0.76–1.34)	1.74 (0.56–5.45)
No	Yes	No	1.18 (0.78–1.79)	2.27 (0.60–8.58)
No	No	Yes	1.08 (0.82–1.43)	2.04 (0.72–5.80)
Yes	Yes	No	1.32 (0.91–1.92)	0.81 (0.10–6.76)
Yes	No	Yes	1.28 (0.92–1.79)	3.98 (1.16–13.60) *
No	Yes	Yes	1.36 (1.09–1.69) *	1.28 (0.54–3.02)
Yes	Yes	Yes	1.29 (1.14–1.46) ***	1.58 (0.93–2.66)

S1: During pregnancy, S2: From birth to one year, S3: From one to three years, Subgroup1: Children with normal birth weight, *n* = 24,027, Subgroup2: Children with low birth weight, *n* = 944, AOR: Adjusted odds ratios. ^a^ Adjusting for children’s gender and age, parental education level, family monthly income, parental age at childbirth and electronic screen exposure. * *p* < 0.05; *** *p* < 0.001.

**Table 5:** Sensitivity analysis of association between astigmatism and combinations of pre- and post-natal ETS exposure among children with a full-term pregnancy and premature infant.

ETS Exposure			Subgroup 1 AOR ^a^ (95% CI)	Subgroup 2 AOR ^a^ (95% CI)
S1	S2	S3		
No	No	No	1.00	1.00
Yes	No	No	1.03 (0.79–1.35)	1.16 (0.51–2.65)
No	Yes	No	1.27 (0.87–1.86)	2.11 (0.78–5.72)
No	No	Yes	1.10 (0.84–1.44)	0.93 (0.39–2.21)
Yes	Yes	No	1.22 (0.83–1.78)	0.94 (0.28–3.20)
Yes	No	Yes	1.35 (0.98–1.84)	1.56 (0.63–3.83)
No	Yes	Yes	1.35 (1.09–1.67) *	1.37 (0.75–2.50)
Yes	Yes	Yes	1.30 (1.15–1.46) ***	1.20 (0.84–1.72)

S1: During pregnancy, S2: From birth to one year, S3: From one to three years, Subgroup1: Children with a full-term pregnancy, *n* = 25,801, Subgroup2: Premature infant, *n* = 2089, AOR: Adjusted odds ratios. ^a^ Adjusting for children’s gender and age, parental education level, family monthly income, parental age at childbirth and electronic screens exposure. * *p* < 0.05; *** *p* < 0.001.

**Table 6:** The interaction between hereditary factors and pre- and post-natal ETS exposure on astigmatism.

	Crude OR (95% CI)	Adjusted OR ^a^ (95% CI)
ETS exposure	1.49 (1.28–1.72) ***	1.34 (1.16–1.56) ***
Parental history of visual problems	2.62 (2.30–2.97) ***	2.72 (2.39–3.10) ***
ETS exposure × Parental history of visual problems	0.88 (0.73–1.06)	0.88 (0.72–1.06)

ETS: Environmental tobacco smoke. ^a^ Adjusting for children’s gender and age, parental education level, family monthly income, parental age at childbirth and electronic screen exposure. *** *p* < 0.001.

## Supplementary Materials

The following are available online at https://www.mdpi.com/1660-4601/16/19/3725/s1, Figure S1: Directed acyclic graph for the association between environmental tobacco smoke exposure in early life and astigmatism, showing all potential confounders. Pink lines indicate potential confounders, Figure S2: Directed acyclic graph for the association between environmental tobacco smoke exposure in early life and astigmatism, showing only confounders retained in the final models. Pink lines indicate potential confounders.
