# Supplementary material for: Associations between Environmental Tobacco Smoke Exposure in Early Life and Astigmatism among Chinese Preschool Children
Source: Int J Environ Res Public Health. 2019 Oct 3;16(19):3725. doi: 10.3390/ijerph16193725 (PMC6801470; doi:10.3390/ijerph16193725)
Supplement: Supplementary file 1 [file ijerph-16-03725-s001.pdf]

Supplemental Materials

Contents

Fig. S1. Directed acyclic graph for the association between environmental tobacco smoke exposure in early life and astigmatism, showing all potential confounders. Pink lines indicate potential confounders.

Fig. S2. Directed acyclic graph for the association between environmental tobacco smoke exposure in early life and astigmatism, showing only confounders retained in the final models. Pink lines indicate potential confounders.

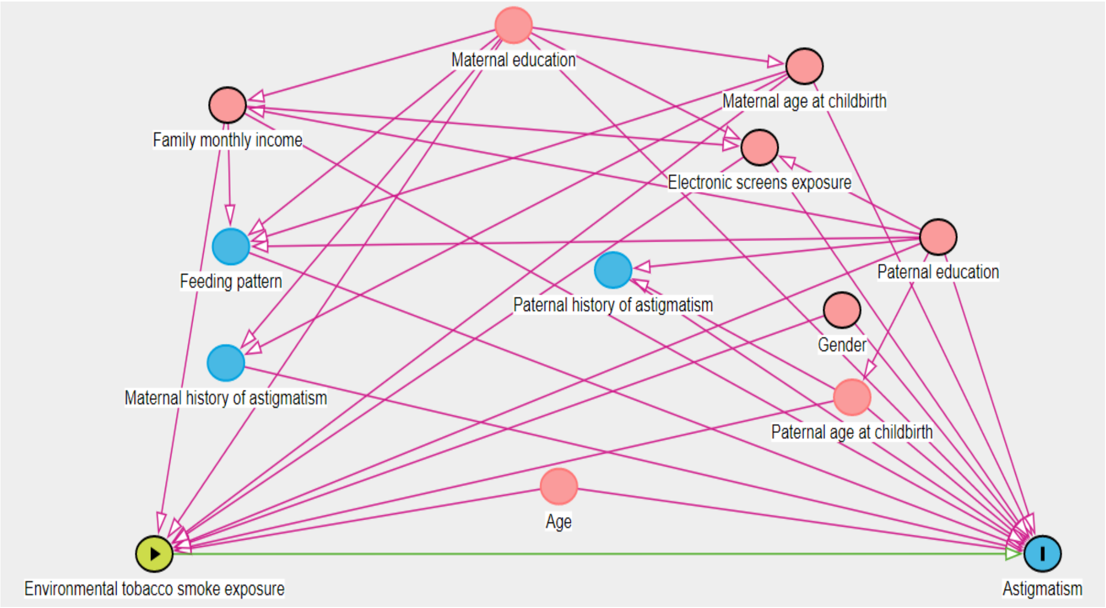

Fig. S1. Directed acyclic graph for the association between environmental tobacco smoke exposure in early life and astigmatism, showing all potential confounders. Pink lines indicate potential confounders.

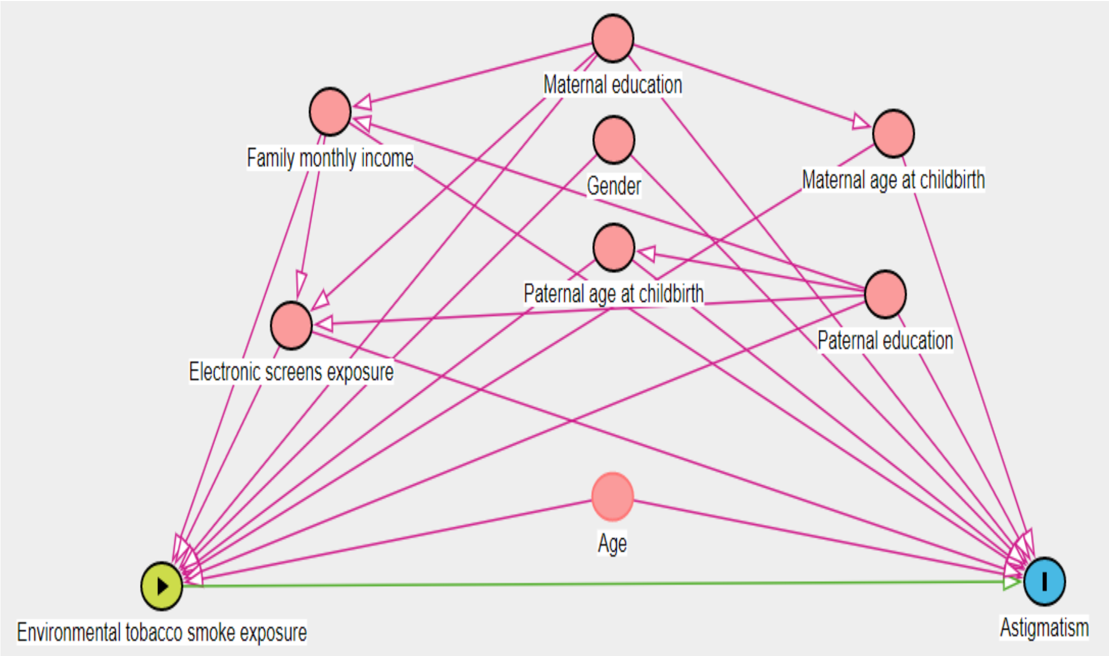

Fig. S2. Directed acyclic graph for the association between environmental tobacco smoke exposure in early life and astigmatism, showing only confounders retained in the final models. Pink lines indicate potential confounders.
